# Comparison of performance of artificial intelligence tools in answering emergency medicine question pool: ChatGPT 4.0, Google Gemini and Microsoft Copilot

**DOI:** 10.12669/pjms.41.4.11178

**Published:** 2025-04

**Authors:** Iskender Aksoy, Merve Kara Arslan

**Affiliations:** 1 Iskender Aksoy Department of Emergency Medicine, Faculty of Medicine, Giresun University, 28100, Giresun, Turkey; 2 Merve Kara Arslan Department of Emergency Clinic, Bulancak State Hospital, 28300, Bulancak, Giresun, Turkey

**Keywords:** Artificial Intelligence, ChatGPT, Copilot, Emergency medicine, Gemini, Medical education

## Abstract

**Objective:**

Using artificial intelligence tools that work with different software architectures for both clinical and educational purposes in the medical field has been a subject of considerable interest recently. In this study, we compared the answers given by three different artificial intelligence chatbots to the Emergency Medicine question pool obtained from the questions asked in the Turkish National Medical Specialization Exam. We tried to investigate the effects on the answers given by classifying the questions in terms of content and form and examining the question sentences.

**Methods:**

The questions related to emergency medicine of the Medical Specialization Exam questions between 2015-2020 were recorded. The questions were asked to artificial intelligence models, including ChatGPT-4, Gemini, and Copilot. The length of the questions, the question type and the topics of the wrong answers were recorded.

**Results:**

The most successful chatbot in terms of total score was Microsoft Copilot (7.8% error margin), while the least successful was Google Gemini (22.9% error margin) (p<0.001). It was important that all chatbots had the highest error margins in questions about trauma and surgical approaches and made mistakes in burns and pediatrics. The increase in the error rates in questions containing the root “probability” also showed that the question style affected the answers given.

**Conclusions:**

Although chatbots show promising success in determining the correct answer, we think that they should not see chatbots as a primary source for the exam, but rather as a good auxiliary tool to support their learning processes.

## INTRODUCTION

Today’s developing technology will result in the use of artificial intelligence (AI) in medical education as in all areas of life.^1^ AI terminology, which started to be used in the 1960s, has taken on a structure that can make work easier and play a role in critical processes today.^2^ Studies that try to reveal the effectiveness of ChatGPT, the most current version 4.0, has the potential to reveal innovations in many areas, from helping us with discharge for patients to summarizing the literature on a certain subject, answering questions and providing information on ethical issues.^3^ Studies show that, in addition to ChatGPT, derivatives such as Google Gemini and Microsoft Copilot are also chatbots whose quality is widely evaluated in the field of medicine. ChatGPT 4.0 and Copilot use the GPT (Generative Pre-trained Transformer) architecture, while Gemini uses LaMDA (Language Model for Dialogue Application) and PaLM 2 (Pathways Language Model) along with web search.^4^

Emergency clinics often require the fastest and most efficient use of resources and timely decision-making processes despite high patient volumes. Due to the unique nature of this field, interest in the application of AI tools to emergency healthcare has increased significantly.^5^ AI applications also have the potential to overcome challenges with objective and data-driven contributions to these environments and to facilitate processes by minimizing human effects such as psychological fatigue.^6^ Despite all this, physicians should learn to work together with AI instead of surrendering to it, and interpret the outputs in the clinical context of patients.^7^

So can these chatbots be considered as a reliable source in the information acquisition processes of emergency medicine residents and candidates? Considering the increasing prevalence of AI in medical education, its role as a consultant and its use in complex situations, it has become frequently used by medical school students, interns, residents and specialists.^8^ In this study, we compared the performance of three different chatbots, ChatGPT 4.0, Gemini and Copilot, in answering these questions by creating an emergency medicine question pool from the national medical specialty exam (MSE) questions, which is an important step in the transition from general practice to specialization in Turkey. We consider the answers given from different perspectives according to both the content and quality of the question.

## METHODS

The aim of the study was to determine how much AI programs can help emergency physicians in patient diagnosis and treatment. In order to measure this aim, the questions of the MSE applied to medical doctors in Turkey were taken as basis. The reasons for this exam being taken as basis are that it was prepared by expert academicians in the field by taking resources from current guides and textbooks. MSE questions that are openly accessible on the official website of the Measurement, Selection and Placement Center (MSPC) were used (https://www.osym.gov.tr/TR,15072/tus-cikmis-sorular.html).

### Ethical statement:

Ethics committee approval was not obtained as the study did not involve an experiment or survey on humans, which requires ethics committee approval.

### Inclusion criteria:

- Questions between the years 2015-2024 were evaluated in order to include the current status of the medical literature.
- The questions were examined by two physicians who are experts in emergency medicine, and the questions related to emergency medicine were recorded.
- Free versions of ChatGPT-4, Gemini, and Copilot were used without logging in.

### Exclusion criteria:

- Questions containing visuals such as figures or pictures were excluded.
- Questions related to the process after applying to the emergency service and being hospitalized in case-type questions were not included in the study.

Before the questions were asked, the text section of the programs was written as “I am a faculty member in the Department of Emergency Medicine at the Faculty of Medicine. I will ask you multiple choice questions in the field of Emergency Medicine and I will ask you to tell me which answer is correct and which is the answer choice.” The answers given to the questions were recorded. The answers given were compared according to the answer key on the MSPC website. Since the programs may have a memory retention feature, a new chat was opened for each question. The word counts of the questions and question types were recorded. The relevant subject areas and subheadings of the questions that the AIs got wrong were noted.

### Statistical analyses:

Statistical analyses were performed using IBM SPSS v23. The Mann Whitney-U test was used to compare quantitative data. Qualitative data were compared using the Pearson Chi-square test. Data were presented as median (minimum–maximum) and n (%). Statistical significance was accepted as p<0.05.

## RESULTS

A total of 248 questions related to emergency medicine clinics were included in the study between 2015-2024. Eleven questions were removed from the study because they were related to emergency but contained visual content. Gemini gave the most wrong answers with 22.9%, followed by ChatGPT with 15.9%. Copilot AI gave the least wrong answers with 7.8% (p<0.001) (Table-I). It was not statistically significant whether the questions were case questions or contained simple information. In order to compare the difference between the length of the questions and the correct answer, the number of words in the question part and the answer part of each question were noted. When both the question part and the answer part were evaluated separately and as the total question text, no significant difference was found (Table-II).

**Table-I:** Question answering rates of chatboxes.

	ChatGPT	Gemini	Copilot	p
True	217 (84.1)	199 (77.1)	238 (92.2)	<0.001*
False	41 (15.9)	59 (22.9)	20 (7.8)	

* Difference between ChatGPT-CoPilot and Gemini-CoPilot.

**Table-II:** Comparison of question type and question length with chatbox answers.

	ChatGPT			Gemini			Copilot		
	True	False	p	True	False	p	True	False	p
Information	75 (81.5)	17 (18.5)	0.398	66 (71.7)	26 (28.3)	0.125	84 (91.3)	154 (92.8)	0.673
Case	142 (85.5)	24 (14.5)		133 (80.1)	33 (19.9)		8 (8.7)	12 (7.2)	
Word Count	56 (13-134)	58 (14-119)	0.921	58 (13-134)	54 (14-119)	0.431	56 (13-134)	56 (16-111)	0.973
Question part	43 (7-108)	38 (7-108)	0.486	45 (7-108)	38 (8-108)	0.197	43 (7-108)	47 (9-81)	0.903
Answer part	13 (5-76)	13 (2-61)	0.739	12 (2-76)	13 (5-61)	0.081	13 (2-76)	10 (5-61)	0.367

The questions that the AIs made wrong were noted as medical subheadings and types of questions. ChatGPT and Gemini made the most mistakes in the fields of trauma, surgical approach and toxicology, while Copilot, which answered the least wrong questions, made the most mistakes in surgical approach and trauma questions. The most common type of question that was answered incorrectly by the AIs was the ‘most likely (diagnosis/treatment/approach) answer’ type. For ChatGPT and Gemini, the second most frequently answered incorrect question type was the ‘most appropriate answer’ type, whereas for Copilot, it was the ‘which option is correct’ type. While AIs gave wrong answers to 11 (4.3%) questions in total, the most wrong answers occurred in “most likely answer” type questions. Only two AIs gave wrong answers to 22 (8.5%) questions, while only one AI gave wrong answers to 43 (16.7%) questions. Copilot made no mistakes in gastroenterology, chest diseases, hematology, cardiology subheadings and question asking techniques such as ECG, classification and definition. While Gemini did not give wrong answers to questions related to gynecology, ChatGPT did not give wrong answers to question types that included classification and definition. All three AIs were notable for giving wrong answers to pediatrics and burns (Table-III).

**Table-III:** Analysis of incorrect answers of chatboxes.

		ChatGPT	Gemini	Copilot	At least one AI give false answer	At least two AI give false answer	All AI give false answer
Surgery		7 (17.1)	9 (15.3)	5 (25)	8 (18.6)	2 (9.1)	3 (27.3)
	General Surgery	6 (14.6)	8 (13.6)	4 (20)	5 (11.6)	2 (9.1)	3 (27,3)
	Gynecology	1 (2.4)	0 (0)	1 (5)	2 (4.7)		
Internal medicine		14 (34.1)	19 (32.2)	6 (30)	17 (39.5)	5 (22.7)	4 (36.4)
	Electrolyte disorder	1 (2.4)	4 (6.8)	3 (15)	3 (7)	1 (4.5)	1 (9,1)
	Endocrinology	1 (2.4)	0 (0)	0 (0)	1 (2.3)		
	Infection	1 (2.4)	3 (5.1)	1 (5)	2 (4.7)		1 (9,1)
	Gastroenterology	2 (4.9)	2 (3.4)	0 (0)	4 (9.3)		
	Pulmonology	2 (4.9)	2 (3.4)	0 (0)		2 (9.1)	
	Hematology	2 (4.9)	2 (3.4)	0 (0)		2 (9.1)	
	Nephrology	1 (2.4)	0 (0)	0 (0)	1 (2.3)		
	Neurology	4 (9.8)	4 (6.8)	2 (10)	4 (9.3)		2 (18,2)
Cardiac		3 (7.3)	6 (10.2)	0 (0)	3 (7)	3 (13.6)	
Pediatrics		4 (9.8)	3 (5.1)	3 (15)	1 (2.3)		3 (27.3)
Toxicology		6 (14.6)	7 (11.9)	2 (10)	7 (16.3)	4 (18.2)	
Trauma		7 (17.1)	15 (25.4)	4 (20)	7 (16.3)	8 (36.4)	1 (9.1)
	Burn	1 (2.4)	1 (1.7)	1 (5)			1 (9,1)
Question type							
Which option is correct		3 (7.3)	4 (6.8)	3 (15)	1 (2.3)		3 (27.3)
Which option is incorrect		6 (14.6)	7 (11.9)	2 (10)	7 (16.3)	4 (18.2)	
Most possible		20 (48.8)	24 (40.7)	11 (55)	18 (41.9)	11 (50)	5 (45.5)
Most appropriate		8 (19.5)	13 (22)	2 (10)	9 (20.9)	4 (18.2)	2 (18.2)
First thing to do		2 (4.9)	3 (5.1)	1 (5)	3 (7)		1 (9.1)
ECG		1 (2.4)	2 (3.4)	0 (0)	1 (2.3)	1 (4.5)	
Calculation		1 (2.4)	3 (5.1)	1 (5)	1 (2.3)	2 (9.1)	
Classification		0 (0)	2 (3.4)	0 (0)	2 (4.7)		
Definition		0 (0)	1 (1.7)	0 (0)	1 (2.3)		
Total		41 (100)	59 (100)	20 (100)	43 (100)	22 (100)	11 (100)

## DISCUSSION

In the study, we found that Copilot was the most successful chatbot in answering emergency medicine questions asked in the Turkish national MSE, while Gemini was the least successful. It was noteworthy that all chatbots had the highest margin of error in questions about trauma and surgical approaches, and that they made mistakes in burns and pediatrics. The increase in error rates in questions containing the root “probability” also showed that the question style affected the answers given. Despite studies examining the accuracy of multiple chatbots in different fields, the limited number of studies in the field of emergency medicine limits the comparison of our study with the existing literature.^9^

AI chatbots show promising results in clinical decision-making processes, clinical inferences, medical education and research development.^10^,^11^ ChatGPT 4.0 is more successful than older versions, especially in clinical case analysis.^12^ In our study, no significant differences were found despite the numerical superiority of ChatGPT 4.0 in correct answer rates in clinical questions compared to the older version. However, Copilot was more successful than both versions of ChatGPT in clinical questions.

The efficiency of these chatbots may depend on model updates and question complexity. Software infrastructures may also affect response times, accuracy, and the possibility of making methodological or logical errors. Contrary to the results in our study, a study comparing these three chatbots found that ChatGPT 4.0 was the most successful tool in questions prepared according to the ERC guideline (with an accuracy rate of 87%) and that Copilot and Gemini had greater margins of error.^13^ Apart from studies reporting that ChatGPT was more successful than other chatbots in topics such as toxicology, triage, and ECG, there are quite a few studies reporting that its use is still limited, especially in triage.^14^-^17^ In another study by Günay et al., it was shown that ECG reading skills were better than emergency medicine specialists. It has been reported that cardiologists still outperform ChatGPT in difficult and complex ECG readings.^18^

Studies demonstrating the superiority of Copilot in areas such as ear, nose and throat, biochemistry, pathology, palliative care were generally studies conducted with ChatGPT version 3.5. No such comparative study has been found in the literature in the field of emergency medicine.^19^-^22^ It was interesting that there was only one burn question in the exam and that all chatbots gave the wrong answer to the question. A more comprehensive evaluation on this subject was presented by Alessandri-Bonetti et al. In the 50-question evaluation of the Advanced Burn Life Support exam, ChatGPT 3.5 and 4.0 were shown to have 86% and 90% success, respectively. Gemini failed the exam with 70%.^23^ The small number of burn questions in our question pool makes our evaluation on this subject impossible.

This study aimed to evaluate which types of questions the AI models made mistakes on, rather than which questions they answered correctly. Since the question pool was based on expert-selected emergency medicine questions from the Turkish Medical Specialization Exam (MSE) rather than a dataset compiled by the authors, we did not conduct a separate analysis of the questions answered correctly.

This study provides a novel contribution to the medical literature by being the first to directly compare the performance of ChatGPT 4.0, Google Gemini, and Microsoft Copilot in addressing a standardized emergency medicine question pool. The findings of this research offer valuable insights into the capabilities and limitations of these artificial intelligence tools in delivering accurate and reliable answers within the context of emergency medicine. From a clinical perspective, these results hold significance as they can inform the integration of AI tools into medical education and decision-making processes in emergency care. By identifying variations in accuracy, response quality, and overall reliability, this study highlights the importance of selecting and validating AI tools before their application in critical clinical settings. This evidence serves as a foundational step toward optimizing the use of artificial intelligence in enhancing medical education, supporting clinical decision-making, and ultimately improving patient care outcomes in emergency medicine.

### Strength and Limitations of the study:

Strengths of the study include the preparation of questions by field-expert academicians in accordance with current guidelines and the technical evaluation of these questions for linguistic and structural coherence. Limitations of the study involve the selection of examination questions from a question pool, which may result in an overrepresentation of certain topics while leading to an insufficient number of questions on others.

## CONCLUSION

To fully exploit the potential of AI in medical education, the reliability and accuracy of AI systems need to be further investigated. Although chatbots show promising success in determining the correct answer, we believe that chatbots should not be considered as a primary source for exams, but rather as a good auxiliary tool to support learning processes.^24^

### Author’s Contribution:

**IA and MKA:** Performed the statistical analysis and editing of the article, data collection and writing, critical review and final approval of the manuscript, and are responsible for the integrity of the research.
